# *CAT, GPX1, MnSOD, GSTM1, GSTT1*, and *GSTP1* Genetic Polymorphisms in Chronic Myeloid Leukemia: A Case-Control Study

**DOI:** 10.1155/2014/875861

**Published:** 2014-11-11

**Authors:** Claudia Bănescu, Adrian P. Trifa, Septimiu Voidăzan, Valeriu G. Moldovan, Ioan Macarie, Erzsebeth Benedek Lazar, Delia Dima, Carmen Duicu, Minodora Dobreanu

**Affiliations:** ^1^Department of Medical Genetics, University of Medicine and Pharmacy Tirgu Mures, 38 Gh. Marinescu Street, 540139 Tirgu Mures, Romania; ^2^Department of Medical Genetics, “Iuliu Hatieganu” University of Medicine and Pharmacy, 6 Louis Pasteur Street, 400349 Cluj-Napoca, Romania; ^3^Department of Epidemiology, University of Medicine and Pharmacy Tirgu Mures, 38 Gh. Marinescu Street, 540139 Tirgu Mures, Romania; ^4^Hematology Clinic 1, University of Medicine and Pharmacy Tirgu Mures, 50 Gh. Marinescu Street, 540136 Tirgu Mures, Romania; ^5^Hematology Clinic 2, University of Medicine and Pharmacy Tirgu Mures, 38 Gh. Marinescu Street, 540139 Tirgu Mures, Romania; ^6^“Ion Chiricuta” Cancer Institute, 34-36 Republicii Street, 400015 Cluj-Napoca, Romania; ^7^Pediatric Clinic, University of Medicine and Pharmacy Tirgu Mures, 50 Gh. Marinescu Street, 540136 Tirgu Mures, Romania; ^8^Laboratory Medicine, University of Medicine and Pharmacy Tirgu Mures, 50 Gh. Marinescu Street, 540136 Tirgu Mures, Romania

## Abstract

Oxidative damage at the DNA level may be promoted by high levels of reactive oxygen species (ROS), leading to genomic instability and increased neoplastic risk. Superoxide dismutase (SOD), glutathione peroxidase (GPX), and catalase (CAT) enzymes are implicated in the prevention of DNA damage by ROS. The aim of the study was to investigate the relationships between *CAT* C262T, *GPX1* Pro198Leu, *MnSOD* Ala16Val, *GSTM1, GSTT1*, and *GSTP1* Ile105Val polymorphisms and the risk of CML. No association was observed between CML and variant genotypes of *GPX1, MnSOD, GSTM1*, and *GSTT1* polymorphisms in any of the investigated cases. Our study suggests that the homozygous variant genotype of the *GSTP1* Ile105Val gene polymorphisms may be associated with the risk of developing CML (OR = 2.5; 95% CI = 1.08–5.7; *P* value = 0.02), while the heterozygous genotype of the *CAT* C262T polymorphism seems to have a protective effect against CML (OR = 0.59, 95% CI = 0.39–0.89, *P* value = 0.01). In most cases, no association was found between laboratory parameters and prognostic factors and the variant genotype of investigated gene polymorphisms. We concluded that *CAT, GPX, MnSOD, GSTM1*, and *GSTT1* gene polymorphisms are not associated with the risk of CML. Variant genotype of the *GSTP1* Ile105Val gene polymorphisms may contribute to the risk of developing CML.

## 1. Introduction

According to Yuzhalin and Kutikhin [1] high levels of reactive oxygen species (ROS) and long-term accumulation of ROS may promote the oxidative damage at the DNA level. This process could affect several genes responsible for the regulation of proliferation, growth, survival, apoptosis, and invasion, leading to genomic instability, deregulation of transcription, induction of mitogenic signal transduction pathways, and replication errors. All these phenomena might finally increase the risk of neoplasia [1]. Several enzymes, such as superoxide dismutase (SOD), glutathione peroxidase (GPX), catalase (CAT), nitric oxide synthase (NOS), and paraoxonase (PON), function to prevent damage caused by ROS.

Manganese superoxide dismutase (MnSOD) catalyzes the conversion of highly reactive superoxide radicals to H_2_O_2_. Hydrogen peroxide is decomposed by mitochondrial peroxiredoxins (catalases or mitochondrial glutathione peroxidases). However, some of the polymorphisms of the* MnSOD* gene lead to an increased superoxide dismutase activity, yielding increased quantities of H_2_O_2_. This process could contribute to the neoplastic transformation of the cells [1]. Such a polymorphism of the* MnSOD* gene (Val16Ala), located in the mitochondrial targeting sequence, has been described to be associated with the overexpression of* MnSOD*, leading to an increased production of hydrogen peroxide (H_2_O_2_), which generates increased levels of ROS, if not subsequently neutralized [2].

Catalase (CAT) participates in defense mechanisms against oxidative stress by converting H_2_O_2_ to water and molecular oxygen [3]. A common polymorphism of the* CAT* gene, namely C262T, has been associated with lower catalase activity and thus increased levels of ROS [2].

The antioxidant enzymes glutathione peroxidases (GPX) are implicated in protection of cells against oxidative damage by reducing H_2_O_2_ and other organic peroxidases to water with reduced glutathione [3]. A polymorphism of the* GPX1 *gene, namely Pro198Leu, has been shown to be associated with malignancies [4]. It was reported that this amino acid substitution decreased the enzymatic activity of glutathione peroxidase [5] in a cell line overexpressing the mutant protein compared with the same cell line overexpressing similar amounts of the wild-type enzyme. Therefore the* GPX1* gene polymorphism may increase the risk of cancer induced oxidative DNA damage.

Glutathione S-transferases (GSTs) enzymes, encoded by* GST* genes, are implicated in detoxification of xenobiotics, and they have a crucial role in protecting tissue from oxidative damage. The variation at the* GSTM1*,* GSTT1*, and* GSTP1* loci has been linked with an increase in the occurrence of cancers, probably due to an increased susceptibility to environmental toxins and carcinogens [6]. The researchers focused on several polymorphisms of the* GST* genes: the homozygous deletions of the* GSTM1* and* GSTT1* genes (the “null genotypes”) and the Ile105Val substitution in the* GSTP1* gene. It has been assumed that these polymorphisms lead to reduced enzymatic activity, thus decreasing their detoxification ability for certain mutagens and carcinogens. This could cause in turn DNA damage and thus a greater risk of developing cancers [7].

To our knowledge, the relationship between the* CAT *C262T*, GPX1 *Pro198Leu, and* MnSOD *Ala16Val gene polymorphisms and the risk of CML has not been previously investigated.

The association between* GSTT1*,* GSTM1, *and* GSTP1* Ile105Val polymorphisms and the susceptibility to chronic myeloid leukemia (CML) was investigated by different studies, but with conflicting results [7–12].

The aim of the present study was to investigate the relationships between* CAT* C262T,* GPX1 *Pro198Leu,* MnSOD *Ala16Val,* GSTM1*,* GSTT1,* and* GSTP1* Ile105Val polymorphisms and the risk of CML.

## 2. Materials and Methods

### 2.1. Patients and Controls

The research protocol was approved by the Ethics Committee of the University of Medicine and Pharmacy Tirgu Mures, Romania, and written informed consent was obtained from controls and CML patients. The study was conducted in accordance with the Declaration of Helsinki.

Our study consisted of 489 individuals from the same geographical area (northwestern and central regions of Romania, comprising a population of around 6.1 million inhabitants), which were divided into two groups: the control and the patients groups.

The control group included 321 healthy individuals without a history of any type of cancer (165 females and 155 males; mean age 47.8 ± 14.4 years).

The patients group was composed of 168 previously untreated adult CML cases (age range 18–80 years; mean age 51.6 ± 13.28 years; 71 females and 97 males) diagnosed at the Hematology Clinic from Tirgu Mures and Hematology Department of the “Ion Chiricuta” Cancer Institute from Cluj-Napoca, Romania, between December 2010 and April 2014. CML was diagnosed based on detection of Philadelphia chromosome by standard cytogenetic and/or* BCR-ABL* fusion gene by reverse transcription polymerase chain reaction.

According to the clinical phases of the disease we included our CML patients into two groups as follows: 141 cases in the group with patients in chronic phase and 27 (16.1%) cases in the group with patients in advanced phase (accelerated and blast crisis).

Additional chromosomal abnormalities (ACA) were found in 22 (13.1%) of our CML cases.

The frequency of ACA in CML patients in advanced phase (accelerated and blast crisis) of the disease was 59% (13 cases).

### 2.2. Genotyping Procedures

Peripheral blood samples were collected in sterile tubes containing EDTA and genomic DNA was isolated using the Quick-gDNA MiniPrep kit (ZymoResearch, USA) and Wizard Genomic DNA Purification Kit (Promega, Madison, WI, USA) according to the manufacturer's instructions.

*CAT *C262T*, GPX1 *Pro198Leu,* MnSOD *Ala16Val, and* GSTP1* Ile105Val genotyping was performed using polymerase chain reaction and restriction fragment length polymorphism (PCR-RFLP) assays as previously described [3, 13–15].

*GSTM1 *and* GSTT1 *polymorphisms were analysed using a multiplex polymerase chain reaction as previously described [16]. 

### 2.3. Statistical Analysis

Statistical analysis was performed using the Statistical Package for Social Sciences (SPSS, version 17, Chicago, IL, USA). Data were considered as nominal or quantitative variables. Nominal variables were characterized using frequencies. Quantitative variables were tested for normality of distribution using Kolmogorov-Smirnov test and were characterized by median and percentiles (25–75%) or by mean and standard deviation (SD), when appropriate. A chi-square test was used in order to compare the frequencies of nominal variables. Quantitative variables were compared using *t*-test, Mann-Whitney test, ANOVA test, or Kruskal-Wallis test, when appropriate. We used the Bonferroni correction or Dunn's correction in order to account for multiple comparisons. The level of statistical significance was set at *P* value < 0.05.

## 3. Results

The frequency of the variant* CAT* 262T allele in CML patients was 22.9%, while in controls it was 27.1% (*P* value = 0.17). The allele frequency of the variant Leu allele for* GPX1* Pro198Leu polymorphism was 50.4% in CML group and 51.5% in control group (*P* value = 0.81). The frequency of the* MnSOD *16Val allele was 54.7% in the patient group and 57.8% in the control group (*P* value = 0.43). The allele frequency of* GSTP1* 105Val was 22.9% in CML patients and 17.4% in controls (*P* value = 0.07). The frequency of* GSTM1* and* GSTT1* null genotypes was 59.5% and 20.8%, respectively, in CML group, and 59.2% and 20.9%, respectively, in the control group (*P* value = 0.94 and 0.99, resp.). In addition we investigated the combined effects of* GSTM1* and* GSTT1* null genotypes regarding the CML risk. There were 18 patients (10.7%) and 45 (14%) controls harbouring both* GSTM1* and* GSTT1* null genotypes, which means a similar distribution (*P* value = 0.37).

When analysing the genotypes of the six polymorphisms genotyped, we concluded that the variant homozygous of the* GSTP1* Ile105Val polymorphism was seen significantly more frequently in patients than in controls (OR = 2.5; 95% CI = 1.08–5.7; *P* value = 0.02).

Interestingly, the* CAT* C262T polymorphism in heterozygous state was found to be protective against CML (OR = 0.59, 95% CI = 0.39–0.89, *P* value = 0.01).

All the genotypes for all other polymorphisms analysed shared similar frequencies between patients and controls (*P* value > 0.05 for all these comparisons). The genotype distribution of the six (*CAT*,* GPX*,* MnSOD*,* GSTP1*,* GSTM1*, and* GSTT1*) gene polymorphisms in patients and controls is presented in detail in Table 1.

In addition we investigated the impact of these polymorphisms in more detail taking into account different factors such as age, gender, phase of the disease (chronic versus blast or accelerated), Hasford and Sokal risk groups, the EUTOS score, the WBC count, blasts, and basophils. No association was observed between the laboratory parameters (blast, basophils, and WBC count) and the* CAT *C262T*, GPX1 *Pro198Leu,* MnSOD *Ala16Val,* GSTM1, GSTT1,* and* GSTP1 *variant genotypes in CML cases.

The* CAT *C262T,* GPX1* Pro198Leu,* MnSOD *Ala16Val,* GSTM1, GSTT1, *and* GSTP1* polymorphisms investigated had no impact on the risk of CML with respect to gender (*P* value = 0.22, *P* value = 0.31, *P* value = 0.68, *P* value = 0.23, *P* value = 0.64, and *P* value = 0.68, resp.) and age (*P* value = 0.24, *P* value = 0.65, *P* value = 0.55, *P* value = 0.60, *P* value = 0.11, and *P* value = 0.59).

None of the polymorphisms* GPX1* Pro198Leu,* MnSOD *Ala16Val,* GSTM1, GSTT1, *and* GSTP1 *was associated with Hasford and Sokal risk groups and EUTOS score in CML patients. In the case of* CAT *C262T polymorphism, it was not associated with Hasford risk groups and EUTOS score, but it was associated with Sokal risk group (*P* value = 0.04) in CML patients.

We analysed the distribution of the six polymorphisms (*GPX1* Pro198Leu,* MnSOD *Ala16Val,* CAT *C262T,* GSTM1 *and* GSTT1* null genotypes, and* GSTP1 *Ile105Val) in patients stratified after the clinical phases of the disease (one group was represented by chronic phase and the other group was represented by accelerated or blast phase). None of the polymorphisms analyzed was associated with the clinical phases of CML (*P* value > 0.05 for all comparisons performed).

Also, we performed a comparison between patients with additional chromosomal abnormalities (ACA) and variant genotypes for the investigated polymorphisms. The frequency of* GSTM1* null genotype was 36.4% in CML patients with ACA and 63% in CML patients without ACA. Our findings suggested that the* GSTM1 *null genotype had a protective effect against the occurrence of ACA (OR = 0.33, 95% CI = 0.13–0.85; *P* value = 0.01).

In the current study, no association was observed between the other five polymorphisms analyzed (*GSTT1* null genotype,* GSTP1* Ile105Ala,* CAT *C262T,* GPX1* Pro198Leu, and* MnSOD *Ala16Val) and the occurrence of ACA in CML patients (*P* value > 0.05 for all comparisons performed).

## 4. Discussion

To our best knowledge, no previous studies have investigated the combined influence of* CAT *C262T*, GPX1 *Pro198Leu,* MnSOD *Ala16Val,* GSTT1*,* GSTM1,* and* GSTP1* Ile105Val variants on CML nor the influence of these genetic polymorphisms on clinical phases of the disease and prognostic factors.

In the present study we observed no significant association between the* CAT *262T,* GPX1* 198Leu, and* MnSOD *16Val variant alleles and the risk of CML (*P* value = 0.17, *P* value = 0.81, and *P* value = 0.43).

It has been reported that the* GPX1* Pro198Leu polymorphism is associated with an increased risk of developing bladder [4, 17], breast [18], and lung [19] cancer, due to a reduction in enzymatic production and activity. No evidence regarding the association between* GPX1* Pro198Leu polymorphism and prostate cancer risk [17] was found.

Taking into account that* MnSOD*,* CAT,* and* GPX1* are involved in the same biological pathways, we analysed a possible synergetic effect of the polymorphisms involving the genes coding for these enzymes. The combined variant (heterozygous and homozygous) genotypes for the* CAT *C262T,* GPX1* Pro198Leu, and* MnSOD *Ala16Val gene polymorphisms revealed no association with the risk of CML (*P* value = 0.17). The frequency of the combined variant genotypes of the* CAT *C262T,* GPX1* Pro198Leu, and* MnSOD *Ala16Val polymorphisms was 33.3% (56 cases) in CML group and 40.05% (105 individuals) in control group.

In the present study, we observed that the* GSTM1* and* GSTT1* null genotypes were not associated with the risk of CML. These findings are in agreement with the study conducted by Löffler et al. [12] on 141* BCR-ABL* positive CML cases and 150 random, healthy blood donors from Germany.

A positive association between* GSTT1* null genotype and the risk of CML was reported by Özten et al. [11], Taspinar et al. [20], and He et al. [8]. The combined* GSTM1* and* GSTT1* null genotypes were significantly associated with the risk of CML [8, 11]. Similar results were reported in a hospital-based case-control study performed on 75 CML patients and 124 unrelated volunteer controls in Kashmiri population [9].

A recent meta-analysis performed by He et al. [8] suggested that GSTM1 polymorphism had no effect on the susceptibility to CML.

We did not observe any association between combined GSTM1 and GSTT1 null genotypes and the risk of CML. Our findings are different compared to those reported by Bhat et al. and He et al. [8, 9]. The conflicting results may be explained by sample size and ethnic background (the studies reported above were made in Asia).

Zhou et al. reported the results of a case-control study conducted on 163 AML cases and 204 controls and found that* GSTT1* null genotype was associated with increased risk of AML in a Chinese population and no association between* GSTM1* null genotype and* GSTP1* Val/Val genotype and AML as independent factors [21].

According to Zhou et al., individuals bearing* GSTM1*,* GSTT1,* and* GSTP1* polymorphisms have a reduced enzyme function. Therefore, they may potentially have an increased cancer susceptibility due to a decreased ability to detoxify carcinogens and reactive oxygen species [21]. Our findings revealed no association between combined* GSTT1* null,* GSTM1* null, and* GSTP1* Val/Val genotypes and CML risk. In addition, we investigated the effects of combined* GSTM1 *or* GSTT1* null genotype with variant* GSTP1* genotypes on CML risk. We failed to find an association between variables mentioned above.

The current study indicated that the presence of homozygous variant genotype (Val/Val)* GSTP1* Ile105Val is a risk factor for CML (OR = 2.5, 95% CI: 1.08–5.7, *P* value = 0.02). Similar results were described in 260 CML cases from Hyderabad, India, by Sailaja et al. [10]. On the other hand, a study from Turkey provided no evidence of a relationship between the GSTP1 IIe105Val polymorphism and susceptibility to CML [7]. Sailaja et al. suggested that CML patients in advanced phase (accelerated and blast crisis) had a higher frequency of heterozygous (Ile/Val) genotype compared to chronic phase [10]. On the contrary, we failed to find an association between variant* GSTP1* Ile105Val genotypes and CML patients in accelerated and blast crisis.

The findings of different studies regarding leukemic risk and* GSTT1*,* GSTM1*, and* GSTP1* Iie105Val polymorphisms are presented in Table 2.

We also studied the combined genotype distribution of the investigated* CAT *C262T,* GPX1* Pro198Leu,* MnSOD *Ala16Val,* GSTM1, GSTT1, *and* GSTP1* polymorphisms in our patients with CML and controls. Our results suggest no effects of the variant combined genotypes of all six investigated gene polymorphisms and the risk of CML. Similar results were obtained in CML cases with three, four, and five variant genotypes from all the combined* CAT *C262T,* GPX1* Pro198Leu,* MnSOD *Ala16Val,* GSTM1, GSTT1, *and* GSTP1* polymorphisms.

In conclusion, our data suggests that* GPX1* Pro198Leu,* MnSOD *Ala16Val,* GSTM1, *and* GSTT1 *gene polymorphisms are not associated with the risk of CML. Our study suggests that the homozygous variant genotype of the* GSTP1* Ile105Val gene polymorphisms may contribute to the risk of developing CML, while the heterozygous genotype of the* CAT *C262T polymorphism seems to have a protective effect against CML.

## Figures and Tables

**Table 1:** Genotype distribution for the six polymorphisms investigated in patients with CML and controls.

Polymorphism	CML patients	Controls	OR (95% CI)	*P* value
	*n* (%)	*n* (%)	CML versus controls	CML versus controls
*CAT* C262T				
CC	105 (62.5%)	168 (52.3%)	—	—
CT	49 (29.2%)	132 (41.1%)	0.59 (0.39–0.89)	**0.01**
TT	14 (8.3%)	21 (6.5%)	1.06 (0.51–2.1)	0.86
*GPX1* Pro198Leu				
Pro/Pro	16 (9.5%)	34 (10.6%)	—	—
Pro/Leu	118 (70.2%)	203 (63.2%)	1.23 (0.65–2.3)	0.51
Leu/Leu	34 (20.2%)	84 (26.2%)	0.86 (0.42–1.7)	0.67
*MnSOD* Ala16Val				
Ala/Ala	1 (0.6%)	1 (0.3%)	—	—
Ala/Val	137 (81.5%)	253 (78.8%)	0.54 (0.03–8.7)	0.95
Val/Val	30 (17.9%)	67 (20.9%)	0.44 (0.02–7.4)	0.53
*GSTP1 *				
Ile/Ile	104 (61.9%)	220 (68.5%)	—	—
Ile/Val	51 (30.4%)	90 (28%)	1.2 (0.79–1.81)	0.39
Val/Val	13 (7.7%)	11 (3.5%)	2.5 (1.08–5.7)	**0.02**
*GSTM1 *				
Present	68 (40.5%)	131 (40.8%)	—	—
Null	100 (59.5)	190 (59.2%)	0.98 (0.67–1.4)	0.94
*GSTT1 *				
Present	133 (79.2)	67 (20.9%)	—	—
Null	35 (20.8)	254 (79.1%)	0.91 (0.56–1.46)	0.70

The bold font means statistically significant, *P* < 0.05.

**Table 2:** Findings of various studies regarding leukemic risk and *GSTT1*, *GSTM1*, and *GSTP1* Ile105Val polymorphisms.

	Association with leukemic risk (OR, 95% CI, *P* value, study)	No association with leukemic risk (OR, 95% CI, *P* value, study)
Combined *GSTM1* and *GSTT1* null genotypes	OR = 9.47, 95% CI = 3.61–24.87, *P* < 0.0001, Özten et al. [11]	—
	OR = 1.79, 95 % CI = 1.24–2.58, *P* = 0.002, He et al. [8]	
	OR = 3.09, 95% CI = 1.12–8.52, *P* = 0.047, Bhat et al. [9]	

*GSTT1* null genotypes	OR = 1.57, 95 % CI = 1.15–2.14, *P* = 0.004, He et al. [8]	OR = 1.34, 95% CI = 0.75–2.40, *P* = 0.32, Löffler et al. [12]
	OR = 2.12, 95% CI = 1.12–4.02; *P* = 0.03, Bhat et al. [9]	
	OR = 3.53, 95% CI = 2.08–6.00, *P* < 0.0001, Özten et al. [11]	
	OR = 2.82, 95% CI = 1.58–5.05, *P* < 0.001, Taspinar et al. [20]	
	OR = 1.64, 95% CI = 1.03–2.63, *P* = 0.03, Zhou et al. [21]	

*GSTM1* null genotypes	—	OR = 0.94, 95% CI = 0.81–1.08, *P* = 0.38, He et al. [8]
		OR = 1.327, 95% CI = 0.73–2.40; *P* = 0.4295, Bhat et al. [9]
		OR = 1.11, 95% CI = 0.69–1.80, *P* = 0.714, Özten et al. [11]
		OR = 0.92, 95% CI = 0.58–1.46, *P* = 0.71, Löffler et al. [12]
		OR = 1.20; 95% CI = 0.78–1.85; *P* = 0.38, Zhou et al. [21]

*GSTP1* Ile105Val genotypes	*P* = 0.0084, Sailaja et al. [10]	*P* > 0.05, Karkucak et al. [7]
		OR = 1.64; 95% CI = 1.03–2.63; *P* > 0.05, Zhou et al. [21].
